# Dr. Adams's Defence of Mr. Hunter's Physiological Ideas and Language, in a Letter to Mr. Clutterbuck

**Published:** 1802-11-01

**Authors:** Joseph Adams

**Affiliations:** Madeira


### *Dr.* Adams's *Defence of Mr.* Hunter's *Physiological Ideas and Language, in a Letter to Mr.* Clutterbuck.

### Letter I.

### To HENRY CLUTTERBUCK, Efq.

Dear Sir,

It gives me much pleafure that you allow me to avail myfelf of this mode of anfwering your printed Letter,* not only on account of the greater facility with which I can offer you my detached thoughts, but becaufe as one part of our controverfy is of the firft importance to the medical world, fo by the prefent vehicle it may be moft univerfally diffufed.

Before I engage to defend Mr. Hunter's language, allow me to obferve with what currency popular opinions are reduced to phrafes, and with how much facility thofe phrafes are received as axioms or aphorifms. Mr. Hunter's little knowledge of language is generally admitted by his friends; but it fhould be remembered, there is much difference between correctnefs and elegance, between clofenefs and eafe. Mr. Hunter's backwardnefs, or, if you pleafe, awkwardnefs of expreffion, arofe often from the clearnefs of his ideas, which rendered him diffatisfied with the common language of Medicine. Before he fubftituted a new term, he was, like all other true philofophers, cautious to examine every exception that could be made againft it; and when he at laft brought it into ufe, we cannot wonder if thofe who were unaccuftomed to its new appropriation, and unacquainted with the train of reafoning which directed him in his choice, fhould at firft feel furprized or diffatisfied: But that his terms were correct, and even his expreffions well chofen, muft be admitted, when we reflect that his language is growing every day more and more the language of Surgery, and his terms and combinations of terms, adopted by thofe who are ignorant of the fource from whence they are derived, and who even undertake to oppofe his opinions. Let me inftance the diftinction *he* firft made between *union by the firft intent* and *adhefive inflammation.* The laft term, now in general ufe, was invented by him from neceffity. He difcovered two diftinct proceffes, which had hitherto been confounded; and to diftinguifh

* See "Remarks on fome of the Opinions of the late Mr. J. Hunter, in a Letter to Dr. Adams of Madeira, Author of Obfervations on Morbid Poifons; by Henry Clutterbuck."

diftinguifh which, of courfe, the language of Medicine did not fupply any term.

Since the invaluable Jennerian difcovery, there is not an expreffion more common than *morbid poifon.* Yet for this neceffary, and as it now appears, obvious diftinction of animal poifons, we are indebted to Mr. Hunter. I will not fay that no difference had been marked between the *hydrophobial* poifon and that of the *viper* ; but you will admit, there was no appropriate term to diftinguifh *morbid* from *original* animal poifons. The confequence was not only an inaccuracy of language, but as muft ever be the cafe, a confufion of ideas: The laws peculiar to one were applied to the other, and remedies and antidotes indifcriminately propofed and adopted.

Let me add, the term fecondary fymptoms, which has now entirely fuperceded the old expreffion of a fecond infection, was firft ufed by Mr. Hunter.

All this appeared to me neceffarily preparative to my defence of Mr. Hunter's diftinction between an *action* in the animal economy and *difpofition to that action :* For, fhould I be fortunate enough to anfwer all your objections, but little would be done, unlefs I could alfo prove the abfolute neceffity of fuch a difcrimination.

Your objections to this application of the word *disposition*, are,

1ft. That Mr. Hunter has not defined the word.

2dly. That it is not new and unappropriated.

3dly. Becaufe it has generally been ufed by medical writers as fynonymous with fufceptibility.

4thly. Becaufe in the fenfe ufed by Mr. Hunter, it muft imply an action. Without fearching Mr. Hunter's ill-connected "Treatife" for a definition, I fhall fatisfy myfelf with tranfcribing my own more at length than you have quoted it.

1ft. That there is a period between the time the infection (of a morbid poifon) is received, and the difeafed action fhows itfelf, is evident in every inftance; and as in moft, we can perceive no alteration during that interval, in the actions of life, this ftate of the conftitution has been called by Mr. Hunter, a difpofition to take on the difeafed action.*

2dly. That the word is not new can be no objection, becaufe every true philofopher, and even philologift, is cautious of coining new words; and when we confider the copioufnefs of our language, it can rarely be neceffary. The coiners of new words,

* Morbid Poifons, p. 49.

words,† and even thofe who have formed antithetic combinations of them, have for the moft part been either fuch as wifh to fport new theories, without meeting thofe objections which would occur if they were ftated in plain language, or elfe fuch as are unwilling to meet an inquiry with its full force. Among the latter I confider Dr. Johnfon's *Obliquity of Rectitude*, which feems to me no better than the holy lies of the Romanifts. But Phyfic abounds with too many inftances of both. Of thefe I have already given inftances enough among our beft received writers. (See Morbid Poifons, chap. i. and ii.)—Without therefore engaging in new controverfies, I fhall only repeat Mr. Hunter's Remarks on Pocky Itch, Rheumatic Gout, &c. all which he very properly confidered as a mere cover for ignorance. When men have any thing to teach, their whole object is to render their language as fimple, and their illuftrations as pointed, as poffible. When they fancy themfelves called upon to form theories, or to explain what they do not underftand, or are too idle to inveftigate, they then have recourfe to quaintnefs of language, novelty of expreffion, and illuftrations which lead the mind aftray rather than direct it towards the object of inquiry.

This I truft will be a fufficient anfwer to your objection, that the word Difpofition was not new; that however, though not new, it was ftill unappropriated, you feem to admit, when you obferve,

3dly. "It has generally been ufed by medical writers as fynonymous with *susceptibility*." If thefe words were formerly ufed as fynonymous, and Mr. Hunter affigned to each a diftinct meaning, he did no more than other philofophers have done; as fuch have appeared to reduce their own fcience to certain laws. It is enough for me to inftance one: Linneus, in his Philofophia Botanica obferves, *Petiolus Pedunculus*, *Pediculus*

† It is remarkable, that Mr. Hunter, though the whole of his Chirurgical Pathology was new, never coined more than one word, and that word he was prevailed upon not to ufe in print. It is well known how much he laboured to reduce the phenomena of fympathy to certain laws. In difcuffing the fubject, and in defcribing the appearances, he was often obliged to repeat the expreffions,—*parts that sympathize*, and *parts originally affected*. To avoid this perpetual periphrafis, he wifhed to fubftitute fingle words. For the firft he felt himfelf authorifed to ufe *Sympathizer*; for the latter, in his Lectures, he coined the word *Sympathent*. This was more a matter of neceffity than choice; becaufe it frequently happens that the fympathizer becomes the difeafed part, while it is difficult to difcover any difeafe in the fympathent, as in gonorrhœa, from dentition, and many other cafes.

*Pediculus antecessoribus fuere synonymi, nobis minime.* In fhort, every man's words are more precife than his predeceffor's, in proportion as his ideas are more correct; and every language is more correct, in proportion as its writers are better informed. Hence the fuperiority of the French in every thing relating to Metaphyfics, and our fuperiority over the whole world (backward as we ftill are) in whatever relates to Pathology.

Your laft objection is, that, "Difpofition in the fenfe ufed by Mr. Hunter muft imply an action."

That this may be the cafe I have already admitted, but it is not lefs certain that it does not imply that action which conftitutes the difeafe.* This would be of lefs confequence if it were an action that would yield to the fame remedies as cure the difeafe. But this is anticipating a queftion which forms the moft important part of Mr. Hunter's doctrine, and includes almoft the whole of our controverfy; I fhall therefore referve it to be difcuffed in a future letter, and at prefent confine myfelf to a few inftances, in which the term *disposition*, as applied by Mr. Hunter, appears to me a neceffary diftinction.

Whenever any new action is neceffary in the animal economy, fome preparatory action is generally to be traced. In many inftances this preparatory action may be traced fo correctly that we might fay, without any violence of language, that the parts are difpofing themfelves for the new action which is to follow. Thus when the tefticles defcend from the abdomen, the peritoneum is elongated at the proper opening, fome time before the tefticle arrives. In parturition the os externum is enlarged, and a proper mucus fecreted often before the os uteri dilates. In difeafed actions the fame *disposition* may be traced. When matter from an abfcefs is approaching the fkin, the cuticle is elongated before ulceration has taken place, and frequently ulceration commences before matter arrives at the furface. When mortification takes place, it is preceded by a coagulation of the blood at the extremities of all the arteries leading to the mortified part.

In thefe inftances, a *disposition* of the parts for the fucceeding action is obvious to our fenfes; the application therefore of the term feems perfectly confiftent with etymology, if that were neceffary.

In conftitutional difeafes, the conftitutional difpofition can only be known by comparing the different phenomena with the order in which they have occurred.

I would firft remark, that in the illuftrations I have offered,

---

* See Morbid Poifons, p. 54.

fered, two different ſeries of actions are pointed out; firſt, a ſet of actions which, though different from the cuſtomary order of the economy, are perfectly healthy and neceſſary for the continuance of the ſpecies; and next, a ſeries of actions which would never take place without a previous diſeaſe. In the firſt therefore the diſpoſition may take place, and the action never follow: For the elongation of the peritoneum takes place even though the teſticle ſhould never reach the opening; and in extra-uterine caſes the enlargement of the os externum and ſecretion of mucus take place, though the fœtus can never arrive at that part. But as far as we can trace diſeaſed parts, the action invariably follows the diſpoſition, and with no leſs certainty the diſpoſition precedes the action. We well know that matter never finds its way to the ſkin without this previous elongation of the cuticle; and when this pointing, as it is uſually called, appears, no prudent ſurgeon ever expects abſorption, or attempts an opening at any other than the elongated part. With the ſame certainty coagulation at the extremities of arteries always precedes mortification; and as far as our experience authorizes us to determine, mortification invariably follows ſuch coagulation, unleſs prevented by the death of the whole ſubject.

Having now illuſtrated the term Diſpoſition in general, as preparatory to a new action which is to take place in a part, or the conſtitution, let us apply it to morbid poiſons.

That there is a period between the application of a morbid poiſon, and the diſeaſed action that follows, requires no proof, and in many inſtances that period has been reduced to laws which vary as little as any phenomenon in pathology. In the ſmall-pox taken by effluvia, the period is about twelve or fourteen days. Under inoculation from eight to ten. In both caſes the patient may be removed from every ſource of infection after the firſt expoſure or inoculation, yet about the uſual time the diſeaſe makes its appearance.

You will perhaps tell me, that in theſe caſes we can trace no conſtitutional action that ſhould induce us to ſuppoſe any *diſpotion* is making for the diſeaſe that is to follow. Give me then another word more appropriate, and I will cheerfully adopt it; but a word I muſt have for the ſtate of a conſtitution which I know to be under the influence of an infection which at a certain period will produce a ſpecific action. Nor is it always true that no preparatory action can be traced during the period which precedes the action of the diſeaſe. Dr. Jackſon, when remarking the ſpace of time between the application of miaſma, and the appearance of fever, obſerves, "the perſon often languiſhes for days, weeks, or even longer. The indiſpoſition

ſuddenly

ſuddenly vaniſhes, and the apparent recovery is ſoon followed by a regular paroxyſm of fever.*

But this diſpoſition to a contagious diſeaſe might be confounded with the diſeaſe itſelf, without danger though not without impropriety, did not experience ſhow us, that remedies which will cure the diſeaſe, have no effect on the diſpoſition, or even in preventing the diſeaſe after ſuch a diſpoſition is formed.

The attentive author above quoted, when ſpeaking of relapſes in intermittent fever, obſerves, that they were not to be prevented by any continuance of the bark immediately after the regular paroxyſms had been ſuppreſſed, but that it was neceſſary to exhibit that remedy ſometimes on the 5th, at others on the 11th, 19th, or 27th day, according to the type the diſeaſe aſſumed in different ſeaſons. Dr. Fordyce makes the ſame remark of our Engliſh intermittents.†

But the moſt important inſtance of all, and that to which the whole of the preceding reaſoning muſt be conſidered as introductory, is, Mr. Hunter's diſcovery of the cauſe why ſecondary ſymptoms of the venereal diſeaſe ſometimes occur after repeated exhibitions of mercury in every known form, and why in many inſtances no ſecondary diſeaſe follows, though the remedy has been no further applied than to cure the firſt local ſymptoms.

In my next I ſhall prove that our beſt authors, ſince the diſeaſe has been accurately deſcribed, have admitted this fact; and ſhow how unſatisfactory every ſolution except Mr. Hunter's has proved, after which I ſhall conſider the theory contained in your letter. I am, &c.

*Madeira, July* 21, 1802. JOSEPH ADAMS.

---

### Letter II.

### To HENRY CLUTTERBUCK, Eſq.

Dear Sir,

HAVING ſaid all that appears to me neceſſary in defence of Mr. Hunter's diſtinction between the ſyphylitic action, and the diſpoſition to take on that action, and left thoſe who diſapprove the expreſſion to favour the world with a better; let us now examine whether the facts which rendered ſome change of language neceſſary have not been admitted by every accurate writer; whether Mr. Hunter has done more than reduce thoſe facts to order; and whether a conſiderate attention to his doc-

* Diſeaſes of Jamaica, p. 134. † Practice of Phyſic.

trine might not have leſſened a late controverſy, which, in comparifon with Phyſic, has rendered the glorious uncertainty of the Law almoſt mathematical evidence.

Having already laboured through volumes, to which my preſent ſituation does not allow me acceſs, and finding myſelf hitherto uncontradicted in my inferences from them,* you will not require me to go further back than Boerhaave. Let me however ſlightly glance at Hoffman and Sydenham. Both of theſe writers acknowledge themſelves unable to prevent the re-appearance of the diſeaſe in ſome form, by any courſes of mercury, with whatever perſeverance, and in whatever manner it was applied; yet both found it curable by mercury when it did re-appear, though each aſſigned different cauſes, and propoſed different forms.

The induſtry of Boerhaave taught him to make more than a verbal diſtinction between *luem eo tempore dominantem et mala venerea quæ latitant.* He aſſerts indeed, that in the ſoft parts he could cure both by mercury, but profeſſes himſelf unable to ſecure the bones by the ſame means, ſpeaking of the loſs of noſes, palates, and other bone caſes, as trophies which diſgrace the whole profeſſion.†

Aſtruc, whoſe accuracy and diligence far exceeded all his predeceſſors, is forced to acknowledge that he knew not how to prevent the re-appearance of the diſeaſe. He ſhows indeed that Boerhaave is miſtaken, in ſuppoſing that mercury will not cure the bones as well as the ſoft parts; but not being aware of Boerhaave's diſtinction between the latent and the apparent diſeaſe, he never meets his adverſary on fair grounds.

Turner acknowledges that he knows of no ſecurity againſt a ſecond infection; but hopes, that if nothing of the kind occurs after a double ſolſtice, we ſhall hear no more of the complaint.

Chapman, like too many other writers, refers the re-appearance of the diſeaſe to bad treatment, but gives no rules by which it can be prevented.

I ſhall not now repeat Dr. Swediaur's language, which forms the whole of his theory. Leaving therefore *radical exterminations*, *retropulsions*, *&c.* to ſuch as can underſtand them, it is enough to obſerve, that he wiſhes for a touchſtone to diſcover the exiſtence of the poiſon in the body, acknowledging that his own experiments have been hitherto inconcluſive.

It is not to be wondered if practitioners ſought for new remedies

---

* See Morbid Poiſons, and the various periodical critiques upon them.
† Prefatio ad Luiſin.

remedies to prevent the recurrence of a difeafe, when their want of candour to each other, or of confidence in themfelves, induced them to refer fuch recurrence to the bad treatment of the firft fymptoms. But after Mr. Hunter had marked a feries and order which feemed to reconcile difficulties admitted by all, though explained by none, one might have expected a more frequent reference to his doctrine on the introduction of a new remedy. Yet we ftill find fome writers furprized at the recurrence of fecondary fymptoms, others urging their great fuccefs in curing thofe fecondary fymptoms, and others minutely relating their fuccefs in curing fymptoms that refifted mercury. Do not fuppofe it is my intention to undervalue thefe writers. Among them is one whofe accuracy is equal, if not fuperior, to any medical writer now living. Whilft therefore we may learn from fuch an example how much is to be done before we meet the public eye, we may be at leaft allowed to wifh that, before he was prevailed upon to add the dignity of his name, he had ufed his cuftomary caution. A reference to Mr. Hunter, would have fhown him that fecondary fymptoms are much more eafily cured than primary ones, and that thofe difeafes, particularly of the bones, which refift mercury, are more commonly the effect of that remedy than of lues.

With the errors of all of us before you, with a critical inquiry into Mr. Hunter's opinions, you have ftarted a new doctrine, which we muft now examine. But let us firft, as all other controverfialifts ought, afcertain what we are to difpute about. About the venereal difeafe, its fymptoms, and cure. What then is a chancre? and what are fecondary fymptoms? You tell me, the inftances I refer to in Celfus, as probably morbid poifons, are nothing more than phagedænic ulcers; but have not other authors defcribed the fame as venereal? and does not their exiftence before that difeafe was known, prove they were not fuch? Of the five firft cafes contained in your letter, you fay "the fymptoms could be traced diftinctly to a venereal origin." My dear fir, learn to be more fceptical. It does not appear by your hiftories, that you faw the primary fymptoms of more than one of the fubjects; how, therefore, are we to afcertain the origin?

As to ulcerated throats, they appear under every circumftance of conftitution, and like other ulcers in which no venereal fource can be traced, when become chronic, are often cured by the new action mercury excites in the part or in the fyftem, or during the convalefcent ftate which follows the mercurial irritation. But I wifh to make as few remarks on cafes, as poffible. Your's are before the public, to whom we both appeal. I have offered only one,* and that does not reft on my own authority. Not becaufe I am fearful my credit fhould be fufpected, but becaufe I am fearful of my own partiality to preconceived notions. In this cafe you conceive all the fubfequent mifchief arofe from fufpending the ufe of mercury; yet it was continued after the difeafe was exafperated in proportion as the fyftem was more affected by mercury, and until there appeared danger that the whole penis would ulcerate away. Other cafes you conceive to be venereal, which yielded to fo little mercury, that none of its effects were perceptible in the conftitution; and at laft you believe that the venereal difeafe, in fome of its ftages, and under certain circumftances, may get well without mercury or any other remedy. If then mercury (as no one will difpute) cures other complaints, and the venereal may get well without mercury, the fuccefs of that remedy can never be confidered as any argument of the reality of the difeafe. This leads us to the former queftion. What is a chancre? I am told a phagedænic ulcer, a floughing ulcer, an ulcer which will yield to mercury, one which will not yield to mercury, and one which will cure itfelf. Such are the primary fymptoms; the fecondary are not lefs uncertain. Can we wonder then if mercury cures all the venereal difeafes which fall under fome practitioners, if acids cure all thofe which are treated by others, or if both remedies exafperate fuch as fall under other hands.

If I could for a moment fuppofe you were afking me where we are to meet with a true defcription of the difeafe, I fhould anfwer, that as far as defcription can convey a true idea, the chancre is accurately traced by all thofe authors whofe works retain any celebrity, and alfo that it is the only kind of ulcer of which Celfus makes no mention, in the paffage to which we both refer. Sydenham, Aftruc, and Hunter, all take notice of the hard edge and bafe of the chancre, and the French writers have given it that name, inafmuch as it was found incurable by the common efforts of the conftitution or by common remedies. Hunter is by far the moft circumftantial in tracing its progrefs, well aware of the neceffity of accurate detail, and alfo as his nice obfervations on every kind of ulcer enabled him to account for the peculiarity of this.

But you will tell me chancres affume different appearances: In their commencement, they will often be affected by any peculiar conftitution of the fubject or air. They will fometimes flough,

* Morbid Poifon, p. 70, & alibi. As the iffue of this cafe was not known when the work appeared, and as in its unfinifhed ftate it attracted the curiofity of feveral medical people, the fequel may make the fubject of a future paper, if the Editors think proper to receive it.

flough, or the furrounding inflammation will be confiderable. But thefe fymptoms fubfide by common remedies or common care, and the true character of the difeafe fhows itfelf. Had all the cafes in which opium, farfaparilla, or acids were tried, been felected with a proper attention to this defcription, we fhould have known the powers of each. Examine the controverfy concerning the firft, and tell me if you can find an accurate defcription of the chancre. Sir W. Fordyce's farfaparilla cafes were moft of them fuch as had refifted mercury. The *acid* controverfy is too recent for me at this diftance to engage in. But I will acknowledge that when I read from authorities I cannot doubt, that *all* the cafes which occurred in a military hofpital for fome months were treated with fuccefs, I find it as difficult to fuppofe none of them were venereal, as that acids will cure the difeafe. To me the controverfy appears ftill undecided; nor can I help regretting, that in the cafes above alluded to, the firft fymptoms were not defcribed with that accuracy which is obfervable in every part of the hiftory during the ufe of the remedies.

I have faid thus much, to fhow the neceffity of defining a chancre; and till we are agreed in this point it will be in vain to difpute. I proceed, therefore, to the next, in which we underftand one another better, namely, Mr. Hunter's doctrine, that mercury cures the difeafed action, but not the difpofition to that action; or, if you prefer Boerhaave's language, that it cures the difeafe when apparent, but not when latent. To fave time I fhall tranfcribe your fummary of the doctrine, adding a few words between [crotchets.]

1ft. That the venereal poifon being taken into the conftitution becomes univerfally diffufed, and contaminates, at once, all the parts which are [at that time] fucceptible of the venereal action, and that it is afterwards expelled the fyftem along with fome of the excretions.

2dly. That the parts contaminated do not immediately go into venereal action; but that they acquire a new ftate or condition, which is termed by him, *a difpofition to take on the venereal action.*

3dly. That difpofition once formed in a part, neceffarily goes on at fome future period *to action.*

4th. That mercury cures venereal action, but does not remove the difpofition previoufly formed, and which is not come into action.

5th. That although mercury does not deftroy the difpofition already formed, yet that it prevents it from forming.

6th. That although the difpofition continues, it does not go into action during the ufe of mercury.

7th. That the action having once taken place, goes on increasing, without wearing itself out.

8th. That [an order of] parts once cured never become again contaminated from the same stock of infection.

9th. That the matter of secondary ulcers is not infectious.

10th. That the venereal action is as soon destroyed in a large chancre as a small one, mercury acting [exciting action] equally in all its parts.

The addition to your first proposition appeared necessary, because, like many other authors, you seem to confound absorption with contamination (p. 11). Wherever there is an ulcer, loss of substance takes place, and this can only be by absorption. In all cases of chancre, therefore, absorption takes place; but contamination only follows in such parts as happen to be susceptible of such an impression at the time the poison is circulating with the fluids.

In the 8th proposition I have added [*order of*] *parts*, because if Mr. Hunter is right, when the disease is cured in a part, it not only does not appear again, from the same stock of infection in that part, but in that order of parts. Thus, when the chancre is cured, the disease will not appear on the genitals from that stock; when the throat and skin are cured, it will not appear again in any of the soft parts; and when the bones are cured, it will not again appear in them. So that if the disease has appeared in all these parts, and been cured, the patient may be said to be free from all the consequences which can arise from that infection.

Had Boerhaave pursued his own inquiries into nature, instead of hunting after the authorities of others, he would probably have drawn the same conclusions as Mr. Hunter. For in the passage above cited, we find him asserting, that mercury would not only cure *luem dominantem—sed mala venerea quæ latitant in locis per quæ arteriæ rubræ, &c. cum idonea velocitate ruunt—perfecte sanari mercurii virtute posse.* That is, it would cure the *apparent* as well as the *latent* disease in the soft parts, though it would not protect the bones. It is evident enough that he first formed this opinion by finding, that after curing the secondary symptoms in the skin, the disease, though it never returned to that part, would afterwards appear in the bones in spite of all his attempts. An unfortunate bone case, which after mercurial courses, was cured by mezereon, led him to suppose, that mercury had no power over the bones whatever. It is hardly necessary to read Astruc's answer, to convince ourselves of Boerhaave's error; but the source of his mistake can only be accounted for on Mr. Hunter's principles.

I am not offering this as an authority, but a medico-historical critique critique which may relieve us both. The truth of Mr. Hunter's doctrine can only be afcertained by experiment. Whilft you are afraid of leaving an ulcer in the throat (of near a month ftanding) without immediately exhibiting mercury, it will be in vain to appeal to your own experiments: mine can only reft on my own authority; and I have feen nothing fince I publifhed, to induce me to alter my opinion. However, the teftimonies above cited from fo many authors, not only prove that they knew no means of preventing the recurrence of the difeafe, but may anfwer your objections to modern practice, and fhew that fecondary fymptoms were probably as numerous formerly as they are at prefent; that they were much more fevere is univerfally admitted; and it is difficult to account for this by any other means than the greater feverity of their mercurial courfes. However, it is fome fatisfaction to find, that our practice need not materially differ, though you are not difpofed to admit Mr. Hunter's doctrine.

In page 17, you remark, the effect of mercury will probably depend, like that of other ftimuli, on the irritability of parts; this irritability, you add, is much increafed by inflammation or ulceration. "Venereal tumours and ulcers are thus more fufceptible of the mercurial irritation than parts whofe fenfibility and irritability remain unchanged, which is the cafe for fome time after contamination. A reafon may hence be afforded why the fame mercurial courfe, which is fufficient for the removal of the apparent action, may be inadequate to overcome that latent degree of the difeafe, which Mr. Hunter terms *difpofition*."

Now, if you are difpofed to admit all this, and recollect the number of mercurial courfes which have been unequal to the prevention of fecondary fymptoms; and alfo, how readily thofe fymptoms are cured when they appear, no better argument can be offered in favour of keeping up the mercurial irritation only long enough to fecure the permanent cure of the firft fymptoms.

In your cafes, you account for the recurrence of fymptoms in the fame order of parts, by fuppofing an inequality in the action of mercury. When the whole conftitution fhows fymptoms of mercurial irritation, it is difficult to form any idea of a partial action. As you refer me to analogy, I would afk, fince your valuable difcovery of the power of mercury in curing the ill effects of lead;* have you ever found, when the conftitution

* See "Account of a new and fuccefsful Method of treating Affections from Lead, &c." [A work which appears hitherto too little attended to. Ed.]

conftitution was generally affected, one hand was relieved whilft the other retained its paralytic affection.

You fay that the records of Medicine by no means warrant Mr. Hunter's conclufion, that "new poifons are arifing every day, refembling the venereal in many refpects," and wonder that none of them were perpetuated if they ever exifted. I thought I had already given a reafon why they could not he perpetuated. You add, "at leaft fo far as for their hiftory and character to be accurately fixed." Their hiftory and character are fixed with fufficient accuracy, at leaft to diftinguifh them from the venereal. The only error has been in calling them by that general name. In confequence of this, Hill picking up a fymptom from all the different writers, afferts that fivvens is only the venereal difeafe; the fame was once faid of yaws. What then are the records of medicine? When we meet with accurate defcriptions of a difeafe, unaltered by our remedies, we may form our own conclufions. Where, let me afk, fhall we meet with a defcription and hiftory of the Small-pox before Sydenham? Of the ulcerous fore throat, as it is called, before Fothergill? You remark too, that we have no fatisfactory hiftory of yaws; the fame may be faid of fivvens. Dr. Rolla has difcovered, and very accurately defcribed, a morbid poifon, either not before known, or, which is the fame thing, not before attended to; and Dr. Trotter has confirmed the fact. It may be afked, as Sydenham's authority is univerfally admitted, why is it not the fame with Mr. Hunter, if he has afcertained the laws of the difeafe in queftion? Whoever carefully examines Sydenham's Hiftory of the Small-Pox (which I truft, by this time, muft be rather a matter of curiofity than neceffity) will find, that how juftly foever he was admired, he was more generally mifunderftood. It is well known too, that a treatment, in direct oppofition to his, maintained its ground for near a century, when his practice was revived, not out of refpect to him, but becaufe its fuccefs threatened a monopoly of this branch of Medicine.

Such are the records of Medicine! Such the hiftorical treatment of a difeafe, the greateft fcourge to the weftern part of the world! Such the uncertain progrefs of a fcience, the moft important to human life; and the profeffors of which, throughout Great Britain, are not lefs liberal than refpected.

You fay, before the venereal difeafe was known in Europe, we have no accounts of fuch difeafes as Mr. Hunter fuppofes to be derived from new poifons. Mr. Becket, however, in his paper in the Philofophical Tranfactions on the Antiquity of the Venereal Difeafe, proves, that long before either the difcovery of America, or the Siege of Naples, other difeafes were known, originating originating in the genitals, and ſpreading from thence over the whole body; that they were even conſidered as ariſing from impure coition, and ſome of them cured by mercury.

Having now ſaid enough to enable the medical world to judge of the queſtion in iſſue, I ſhall take my leave, till you require a preciſe anſwer to any point I may have overlooked, and which appears to you more deciſive than the reſt, or till you furniſh me with new matter. There are, however, two paſſages in your letter, which I muſt not leave unanſwered. By the firſt, it appears as if Mr. Hunter had contradicted himſelf; by the ſecond, that I had miſquoted an author.

In proving that the diſeaſe will ſometimes cure itſelf, you ſay, (p. 28.) "When purple ſpots appear on the ſkin," Mr. Hunter obſerves, p. 319, "giving it a mottled appearance in this diſeaſe, many of the ſpots diſappear, whilſt others continue and increaſe." This is neither correctly copied, nor is the explanatory note added; I ſhould add, my edition is the firſt, 1786. It is in vain therefore to comment till we agree in our text.

In page 63 of your Letter is the following paſſage:—"The caſes quoted by you from the Edin. Med. Eſſays, (vol. 3.) as inſtances of morbid poiſon different from the venereal, do not appear deciſive. The reſemblance of the ſymptoms to the venereal is ſufficiently great to warrant the referring them to the ſame ſource, and they all yielded to the uſe of mercury. You obſerve, that when the ſymptoms recurred in ſome, they gave way to the ſweating bath; but you have omitted to mention, that a few grains of calomel were given before going into the bath, which was thrice a week, and ſometimes two grains of turbith mineral were added to the calomel."

As you have not referred me to the paſſage in Morbid Poiſons, and as, in turning over the leaves, I have met with no other mention of theſe caſes but at page 82, I can only ſay, "We are both wrong." It is true, I have omitted the calomel and turbith; but I ſay nothing about the patient's being cured by the ſweating bath. However, this is a fair implication: But as I admit many other morbid poiſons yield to mercury, it would have been right to take notice of my other objections, when you ſay, "The reſemblance of the diſeaſe is ſufficiently great to warrant the referring it to the ſame ſource."

My other objections, you will find, are,—1ſt. "That the ulcer in the woman's mouth, which infected the ladies, was cured without mercury.—2d. That the diſeaſe was *remarkable* for its malignancy and *swift progress*." I now add, the appearances, as far as deſcribed, are all different from the venereal. After ſaying this, I again thank you for ſetting me to rights, and doubt not that it was with the beſt intentions.

Your's, &c. JOSEPH ADAMS.

